# 6.A. Workshop: Strategies to build up health system resilience: Legacy of the COVID-19 pandemic

**DOI:** 10.1093/eurpub/ckac129.347

**Published:** 2022-10-25

**Authors:** Organised by: Finnish Institute for Health and Welfare, Tampere University, Trinity College Dublin, University of Copenhagen, EUPHA-HSR, Chair persons: Ilmo Keskimäki (Finland), Johan Hansen (EUPHA-HSR)

## Abstract

Health systems are the central part of infrastructure in societies and their smooth operation is important for the continuation of a wide range of critical operations in any country. The COVID-19 pandemic has actualised questions on health system resilience defined as the system's ability to prepare for, manage (absorb, adapt and transform) and learn from external shocks.. However, economic recessions as well as long- and short-term stresses related to aging, climate change and migration, and most recently a war have underlined that also in Europe the governments need to prepare their health systems to face different types of threats in order to guarantee effective protection to population health in their countries. The importance of the task to strengthen health system resilience has been comprehended at national and international level. International actors including WHO, OECD and World Bank have designed policy advice on strengthening health systems against external shocks, the European Union has launched programmes for preparing for future health emergencies, and individual countries are designing policy and system reforms allowing for health crises. In the workshop we address policies to strengthen health system resilience drawing on the experiences in three countries: Ireland which entered the COVID-19 pandemic with a legacy of the recent economic crisis hitting hard health care financing, Finland which has been implementing a major health care reform concomitantly with the pandemic, and Denmark which offers a case study on organisational learning from the pandemic within the hospital sector and chronic patient care. Based on these three case studies the workshop also contemplates whether learning from shocks is always helpful in terms of system transformation or whether they can also create path dependencies that weaken the systems’ ability to prepare for unknown threats. The workshop will broaden the view by discussing the trends of health system reforms in European countries and considering international advice on system and policy reforms to strengthen health system resilience.

**Key messages:**

• Shocks create opportunities for reform and better performance and challenges through overloaded systems and fast decision-making.

• Assuming all shocks are like the last weakens preparedness and adaptability.

